# *My Activity Coach* – Using video-coaching to assist a web-based computer-tailored physical activity intervention: a randomised controlled trial protocol

**DOI:** 10.1186/1471-2458-14-738

**Published:** 2014-07-21

**Authors:** Stephanie Alley, Cally Jennings, Ronald C Plotnikoff, Corneel Vandelanotte

**Affiliations:** 1 Centre for Physical Activity Studies, School of Human, Health and Social Sciences, Central Queensland University, Building 18, Rockhampton, QLD 4702, Australia; 2 Faculty of Physical Education and Recreation, W1-34 Van Vliet Centre, University of Alberta, Edmonton, AB, Canada; 3 Priority Research Centre for Physical Activity and Nutrition, University of Newcastle, Advanced Technology Centre, University Drive, Callaghan, NSW 2308, Australia

**Keywords:** Physical activity, Intervention, Behaviour change, Web-based, Internet, Video calling, Skype, Coaching

## Abstract

**Background:**

There is a need for effective population-based physical activity interventions. The internet provides a good platform to deliver physical activity interventions and reach large numbers of people at low cost. Personalised advice in web-based physical activity interventions has shown to improve engagement and behavioural outcomes, though it is unclear if the effectiveness of such interventions may further be improved when providing brief video-based coaching sessions with participants. The purpose of this study is to determine the effectiveness, in terms of engagement, retention, satisfaction and physical activity changes, of a web-based and computer-tailored physical activity intervention with and without the addition of a brief video-based coaching session in comparison to a control group.

**Methods/Design:**

Participants will be randomly assigned to one of three groups (tailoring + online video-coaching, tailoring-only and wait-list control). The tailoring + video-coaching participants will receive a computer-tailored web-based physical activity intervention (‘My Activity Coach’) with brief coaching sessions with a physical activity expert over an online video calling program (e.g. Skype). The tailoring-only participants will receive the intervention but not the counselling sessions. The primary time point’s for outcome assessment will be immediately post intervention (week 9). The secondary time points will be at 6 and 12 months post-baseline. The primary outcome, physical activity change, will be assessed via the Active Australia Questionnaire (AAQ). Secondary outcome measures include correlates of physical activity (mediators and moderators), quality of life (measured via the SF-12v2), participant satisfaction, engagement (using web-site user statistics) and study retention.

**Discussion:**

Study findings will inform researchers and practitioners about the feasibility and effectiveness of brief online video-coaching sessions in combination with computer-tailored physical activity advice. This may increase intervention effectiveness at an acceptable cost and will inform the development of future web-based physical activity interventions.

**Trial registration:**

ACTRN12614000339651**Date:** 31/03/2014.

## Background

Physical activity improves physical and mental health, and significantly lowers the risk of non-communicable disease including cardiovascular disease, diabetes mellitus and cancer [1]. It is estimated that individuals who are physically active have a 30% to 50% lower risk of non- communicable diseases and have a 20% to 50% lower risk of mortality than inactive individuals [2-4]. The World Health Organisation recommends 30 minutes of moderate intensity activity on 5 days of the week to receive health benefits and reduce the risk of non-communicable disease [5]. Despite this, more than 50% of Australians fail to meet these recommendations [6] which is estimated to cost the Australian economy 13.8 billion each year in healthcare, loss of productivity, and mortality costs [7]. Hence, there is an urgent need for effective physical activity interventions with a broad reach.

High levels of internet access (e.g. 83% in Australians) make the development and dissemination of web-based physical activity interventions worthwhile [8]. Health behaviour change interventions delivered via the internet have the potential to reach a large audience at low-cost, they are convenient for participants and enable the content to be delivered in a non-confrontational way [9-11]. Although the short-term effectiveness of web-based physical activity interventions is well-established, participant retention and engagement have been identified as a challenge with many web-based interventions reporting high dropout rates or low use of the websites after a period of time [12,13]. As the amount of exposure to the intervention content is strongly linked to behavioural outcomes, low participant retention and engagement may limit the effectiveness of web-based interventions [14,15].

Reviews have shown that successful web-based physical activity interventions have included personalised advice through coaching or computer-tailoring, numerous participant contacts, social support elements, and theoretically-based behaviour change techniques [13,16,17]. Randomised controlled trials have found that web-based interventions that provide some form of personalised advice result in improved engagement and behavioural outcomes compared to interventions providing generic advice [18,19]. Online coaching and computer-tailored advice are effective ways of providing personalised advice in web-based interventions that mimic the advice and support provided in traditional face-to-face counselling sessions, in a way that reduces geographical, time and cost limitations [18,20].

Coaching is defined as facilitating health behaviour change and improving health outcomes through interaction or partnership between a health professional (coach) and an individual client [21]. Online coaching sessions provide personal contact similar to traditional face-to-face counselling. Online coaching sessions are typically delivered through private messages (e-mail, SMS), real time instant messaging (chat) and group forums. Online coaching in web-based behaviour change settings has been found to improve perceptions of social support which is positively associated with behaviour change [22,23]. Counsellor initiated private messages and real time counselling sessions have been found to result in greater weight loss compared to web-based interventions providing information on weight loss only [24-27]. Other methods of delivering social support in web-based interventions with lower time and cost restraints include online peer discussions and provision of an available online coach (“Ask the expert” button). Neither method has been found to be successful at improving behavioural outcomes of the intervention, as few participants have shown to use these features [28]. Although the effectiveness of online coaching is well established, the high time and cost investment in comparison to computer-tailored advice means that they are rarely included in web-based health behaviour interventions aiming to reach a wide audience [29,30].

Computer-tailored advice is more common in web-based physical activity interventions as it can be delivered at a lower cost. Computer-tailored advice is automatically produced using a computer-based expert system that delivers feedback based on participant’s responses to a questionnaire [18]. Computer-tailored physical activity advice is read, printed, discussed and remembered more than generic advice [31]. Furthermore, it is also more appreciated by participants, processed more intently and leads to greater attention compared to generic advice [32]. As such, it is not surprising that it leads to improved health behaviour changes compared to generic health advice [33]. Despite the well-established effects of computer-tailoring, it is unknown if computer-tailored interventions would be more effective with an element of human support.

It appears no web-based physical activity interventions have provided both computer-tailored advice and online coaching simultaneously. It is therefore unknown whether this combined approach improves intervention outcomes. When computer-tailored advice is delivered prior to the online counselling session it can largely reduce the time required from a coach to provide feedback, therefore keeping the time and financial costs to conduct the intervention viable to reach large numbers. In addition the computer-tailored advice may reduce reliance on the knowledge and expertise of the coach. The addition of a brief online coaching session may add further explanation; personalisation and interpretation of the theory-based computer-tailored advice as well as provide a social support element [21,34,35]. Furthermore, advances in internet technology and broadband capacity allow the coaching sessions to be delivered via free online video-calling programs (e.g. Skype) which, unlike online instant messaging or forums, enables the participant to view the coach whilst engaging in a verbal discussion. Psychological counselling over video calling programs is becoming widely used and accepted [36]. Video-coaching facilitates higher engagement, feelings of accountability and social support, and reduces the risk of misunderstandings compared to emails and instant messaging [36,37].

The current study will examine the feasibility, engagement, retention and effectiveness of a computer-tailored web-based physical activity intervention, with and without brief online video-coaching sessions. The findings will guide health promotion professionals in delivering future large-scale web-based physical activity interventions that are effective at engaging participants and producing long-term behaviour changes. More specifically this study will assess the between group differences in physical activity outcomes as a result of receiving computer-tailored advice inclusive of video-counselling sessions, compared to computer-tailored advice alone and a wait-list control group. The secondary analyses will assess between group differences in website engagement (website user statistics and fidelity), retention, participant satisfaction, quality of life, and correlates of physical activity (mediators and moderators). The fidelity and satisfaction with the video-coaching sessions will also be measured to assess the feasibility of this intervention approach.

## Methods/Design

### Participants

Participants will be eligible to participate if they are English speaking adults (over 18 years) who reside in Australia, and do not meet the physical activity recommendations. Participants will need to have an internet connection and a computer processing system efficient enough to watch videos online, in order for an online video-calling program (such as Skype, Google Hang Out or Face Time) to work effectively. Participants will be excluded if they are: non- English speaking, pregnant, under 18 years of age, currently meeting the Australian physical activity guidelines (assessed by a single item, ‘do you currently participate in less than 30 minutes of physical activity on average each day?’), or at risk of injury or ill health from increasing their physical activity (assessed by the Physical Activity Readiness Questionnaire [38]).

### Recruitment

Print and internet advertising will be used to recruit participants. Print advertising will include newspaper advertising in newspapers and posters and leaflets promoting the intervention will displayed in sporting clubs, schools, the university and medical centres. The internet advertising will include free posts on community websites, and Google and Facebook advertisements. All advertisements will direct interested individuals to a specific recruitment page that is part of the intervention website where they can find out more information about the study and download the participant information sheet. If they are interested in registering, individuals will be asked for their contact details and to give their consent to participate via an online consent form. A researcher will then call participants via telephone to assess their eligibility. Participants who are eligible will be randomly assigned to one of the three groups and notified of their log-in details and intervention starting date. Participants will be allocated at random using a computer generated sequence. Group assignment will only be disclosed after participants have completed the baseline assessment.

### Procedure

**Figure 1:**
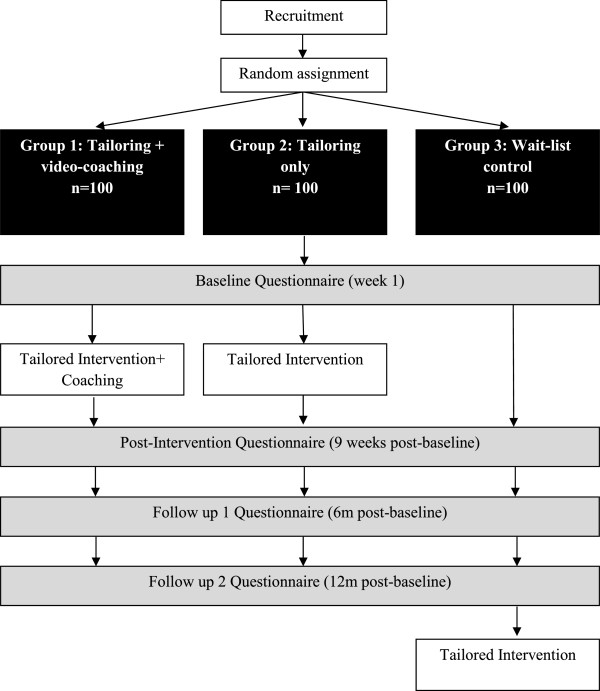
Intervention process.

Participants will be randomly assigned to one of three groups, tailoring + video-coaching, tailoring-only or wait-list control. All tailoring groups will receive a web-based physical activity intervention named ‘*My Activity Coach*’ that consists of 4 modules of computer-tailored advice. Additionally the tailoring + video-coaching participants will also receive 4 brief coaching sessions with a physical activity expert to discuss the personalised advice they received in the previous module. To control for exposure to additional intervention contacts in the tailoring + video-coaching groups the tailoring-only participants will receive a total of 4 tailored emails to remind them of the tailored advice they received in the previous module, but they will not receive any coaching. Questionnaire data will be collected at baseline, immediately post-intervention at week 9, and 6 and 12 months post baseline (see Figure 1). All questionnaires will be completed through the intervention website, including the waitlist control group (though no tailored content will be available for these participants). Satisfaction with the intervention will only be measured at 9 weeks in intervention group participants. Participant retention, engagement, and feasibility of the coaching sessions will be measured for the intervention participants throughout the intervention. Participants in the wait-list control group will be given the opportunity to participate in the intervention after they have completed the 12-month follow-up questionnaire (see Figure 1). The research has been approved by the Central Queensland University Human Ethics Committee (H13/04-044), and complies to the Helsinki Declaration.

### The ‘My Activity Coach’ intervention

The ‘*My Activity Coach’* intervention will provide 4 modules with personalised physical activity feedback over an 8-week period. A new module will become available to participants every second week. In each module participants will log on to the intervention website, complete a brief survey and immediately receive computer-tailored advice based on their answers. Given that all content will be personally–tailored, there will be differences in the information that participants receive. For example, participants who are overweight or obese will receive additional information not provided to participants who are of normal weight, as this information would be irrelevant for them. Photographs of people tailored to participant’s activity levels, age and gender will be included in the feedback. The intervention will also provide participants with an action planning tool to support them in setting detailed physical activity plans during the program [39]. The content of the tailored advice and the action planning tool is described in more detail below. Every second week a new intervention module will become available to participants. The module will appear on the intervention homepage, and participants will receive an email to alert them that it is available. Participants will also receive up to two reminder emails to complete each module if they haven’t already done so. Participants who haven’t completed the module one week after it first became available will receive a reminder phone call. Participants can access and re-complete previous modules up to 12 months post-baseline.

Constructing computer-tailored advice on an empirically supported theoretical framework has been found to improve intervention outcomes [18]. Research has demonstrated that tailoring to a combination of theoretical constructs, behavioural outcomes and demographics is ideal [18,20]. Therefore the tailoring scrips in the current intervention will be predominantly based on one behaviour change theory, Theory of Planned behaviour (TPB) and one communication theory, Elaboration Likelihood Model (ELM). The tailoring scripts will thus tailor to TPB constructs, demographics and physical activity levels [40]. The TPB was chosen as the behaviour change theory to guide the tailored advice as it identifies pathways to behaviour change, has been found to explain a significant amount of variance in physical activity behaviour [41,42] and has successfully been used to guide a number of physical activity interventions over a range of population groups [18,20,43,44]. The TPB [40] proposes that intention is the strongest influence of behaviour, which is in turn influenced by the individual’s attitude, subjective norm, and perceived behavioural control. Attitude refers to the individual’s views on performing the target behaviour, which is formed from assessing the positives and negatives of performing the behaviour. Subjective-norm refers to the individual’s perceptions of how they see their behaviour affecting their significant others. Perceived behavioural control refers to self-efficacy, which is an individual’s belief that they will be able to execute a target behaviour [45], and controllability in performing the target behaviour. Interventions based on TPB target individuals attitudes, subjective-norms and perceived behavioural control to strengthen participant’s intentions to change the target behaviour. Interventions based on TPB also provide tools (e.g., action planning) to facilitate behaviour change arising from intentions [40]. The intervention topics in the *‘My Activity Coach’* program and the corresponding TPB constructs they are designed to target can be found in Table 1.

**Table 1:** Topics, tailoring items and TPB constructs of the computer-tailored physical activity advice

**Module**	**Topic**	**Tailoring variables**	**TPB construct**
**Module 1: ‘Are you active enough?’**	Physical Activity guidelines	None	Attitude
	Normative feedback (also in Graph format), compares participants physical activity to recommendations	Current physical activity levels	Subjective norms
	Physical activity sessions	Current physical activity levels and number of activity sessions each week	Subjective norms
	Importance of physical activity, tailored to current activity levels, BMI and age.	Current physical activity levels, BMI and age	Attitude
	Task self-efficacy	Current physical activity levels, and perceived difficulty with meeting the guidelines	PBC
	Benefits	Top two most important benefits of becoming more active	Attitude
	Suggested goal increase in physical activity	Current physical activity levels	Intention
**Module 2: ‘Let’s set some goals!’**	Feedback on physical activity changes	Physical activity levels at module 1 and 2	PBC
	Coping self-efficacy	Current physical activity levels, and perceived difficulty with meeting the guidelines when not feeling great, busy, and/or do not have an activity buddy	PBC
	Goal setting	Current physical activity levels, and experience and knowledge of goal setting	Intention
	Action plans	Current physical activity levels	Intention
**Module 3: ‘Physical activity and your environment’**	Feedback on physical activity changes	Physical activity levels at module 2 and 3	PBC
	Feedback on progress to meeting action plan	Success at meeting action plan set after module 2	PBC
	Scheduling self-efficacy	Current physical activity levels, and perceived difficulty with scheduling times to get active	PBC
	Utilising physical environment to become more active	Possession of a garden, distance to places regularly visited, working status, length of lunch break and facilities at work.	PBC
	Utilising social environment to become more active	Activity levels of friends and family, support from friends and family, and presence of an activity buddy or sporting team	Subjective norms
**Module 4: ‘Staying active’**	Feedback on physical activity changes	Physical activity levels and number of activity sessions at module 1 and 4	PBC
	Feedback on progress to meeting action plan	Success at meeting action plan set after module 3	PBC
	Barriers	Top two most significant barriers to becoming more active	PBC
	Maintenance self-efficacy	Current physical activity levels, and perceived difficulty with continuing to meet the guidelines	PBC
	Relapse prevention	Physical activity levels at module 1, 2, 3 and 4	Intentions

PBC: Perceived Behavioural Control.

The Elaboration Likelihood Model was also chosen to guide the intervention content to address the formation of participants’ attitudes [46]. The ELM identifies two types of persuasion that influences attitude; central and peripheral. Central persuasion is when an individual takes consideration of ample information to form an attitude. Peripheral persuasion is when an individual allows simplistic associations of negative and positive attributes to form their attitude. Stronger and longer-term attitudes are likely to result from central persuasion. The central persuasive route is likely to occur with high elaboration (including evaluation, recall and judgment) [46]. DD Rucker and RE Petty [47] explain that in order to facilitate elaboration of health promotion messages, interventions need to give listeners enough information about the health behaviour, demonstrate the credibility of the information, make the information relevant to the listener, and repeat the key messages. Therefore *‘My Activity Coach’* participants are provided with information on the specific benefits of physical activity supported by research findings and trusted organisations (e.g. World Health Organisation). The participants are encouraged to see how physical activity is relevant to them, and the key benefits of physical activity and the recommended amount of physical activity are presented in different forms (e.g., text, graph) [47].

### Physical activity progress feedback

**Figure 2:**
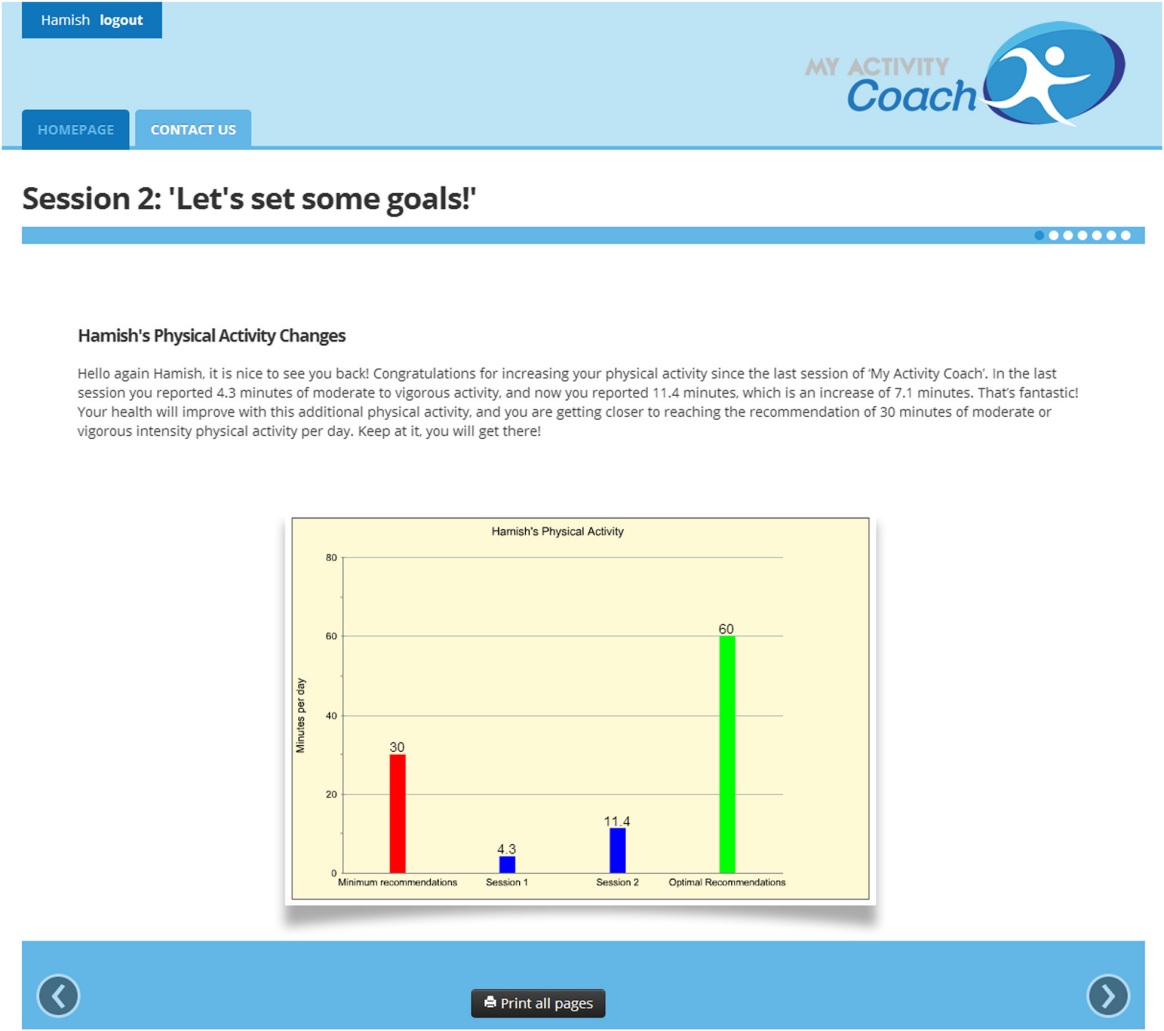
Tailored advice including physical activity graph.

Participant’s physical activity will be assessed via the validated Active Australia Questionnaire (AAQ) in every module. The tailored advice in Module 1 will begin with a graph of participant’s current level of physical activity compared to the minimum and optimal recommendations. The tailored advice in Module 2, 3 and 4 will begin with a graph of participants’ current physical activity, their physical activity at the previous modules, and the minimum and optimal recommendations (see Figure 2). Comparing participants physical activity levels to the recommendations is included to increase awareness of their own activity levels, and emphasising progress over time has been found to improve participants self-efficacy [48]. In module 3 and 4 participants will also receive a tailored statement about their success in completing the action plan they set in the previous module which will include appropriate feedback in creating their next action plan.

*Module 1*, titled ‘Are you active enough’, will cover the importance of physical activity and the physical activity recommendations. Module 1 will introduce participants to the intervention, explain the physical activity recommendations in relation to participants’ current level of physical activity, and explain the health benefits of physical activity tailored to their BMI, age and level of physical activity. Participants will also receive personalised feedback about the benefits of becoming more active. Beliefs of the benefits of physical activity have been found to explain a significant amount of the variation in attitude to becoming more active [49]. Participants will receive a tailored statement addressing their task self-efficacy which is essential for starting exercise [45]. Task self-efficacy refers to participant’s belief that they can meet the physical activity recommendations. The module ends with a suggested goal (based on their current activity level) to work towards until they receive the next module 14 days later. Goals set by researchers have been found to produce higher self-efficacy [48].

*Module 2,* titled ‘Let’s set some goals’, will provide participants with information on goal setting and action planning. Information on creating SMART (Specific, Measurable, Achievable, Realistic and Timely) goals will be provided to participants. Goal setting is acknowledged as a successful strategy in improving physical activity levels and targets participants perceived behavioural control [50]. Azjen recommends that interventions based on the Theory of Planned Behaviour should also include implementations intentions (or action planning) to facilitate behaviour changes resulting from participants intentions to change the behaviour [51]. Action planning requires participants to determine the specifics of how they will reach their goals (e.g., what, where, when, etc.). Action plans have been successful at improving participants health behaviours including physical activity [39,51]. Participants will also receive a tailored statement addressing their coping self-efficacy for common barriers including business, tiredness and lack of an activity partner. Coping self-efficacy is essential for exercise adherence [45].

*Module 3,* titled ‘Physical activity and your environment’, delivers tailored information on utilising participant’s social and physical environments to increase their physical activity. Participants will receive tailored information regarding their physical environment including whether they have a garden, how far they live from places regularly visited, whether they work full time, how long their work lunch breaks are, and if they have showering facilities at work. Participants will also receive tailored information about their social environment including whether they are active with others and whether their family and friends are active and/or support them in becoming more active. Participants will also receive a tailored statement addressing their scheduling self-efficacy which is an important for exercise adherence [45]. For example, participants who indicate that it will be hard to schedule 30 minutes of physical activity every day will be given tips to help them find times to get active (just do three 10 minute walks, or walk to the shops and back, or walk with a friend instead of meeting at the café), to illustrate that it is achievable.

*Module 4,* titled ‘Staying active’, addresses participant’s barriers to leading an active lifestyle and covers relapse prevention. Participants will be given tailored information about their most significant barrier to support them in overcoming them. Participants beliefs about significant barriers to becoming more active has been found to explain a significant amount of the variation in perceived behavioural control [49]. Module 4 will also provide participants with information on relapse-prevention. Relapse prevention helps participants identify specific high-risk situations for relapse, enhances coping skills within those situations, helps participants manage lapses so it doesn’t lead to a relapse, and restructures participant’s perceptions of the relapse process. Research findings support the effectiveness of relapse prevention at reducing participants relapses [52]. Lastly, participants will receive a tailored statement addressing their maintenance self-efficacy. Here participants who indicate it will be difficult to maintain an active lifestyle will be encouraged that it is achievable once habits are formed. Table 1 explains the sections in each module of the personalised activity advice, how the advice is tailored, and the Theory of Planned Behaviour constructs that the section aims to address in order to improve physical activity behaviour.

### Action planning tool

An action planning tool will be provided to guide participants in setting an effective action plan. The action planning tool is made up of a structured form where participants can enter up to 4 different activities they plan to do in the upcoming fortnight. For each activity they will be asked where they will do it, when they will do it, for how long they will do it (session duration), and who will support them. Participants will be provided with information and tips to guide them in choosing their activities, locations, time, and support person. After participants have completed their action plan they will be provided with an overview in the format of a weekly calendar with the times they selected to participate in each of the activities including their support person and the location. Participants are encouraged to print their action plan, and carry it out over the following two weeks. Participants will be encouraged to create an action plan after module 2 (where the concept of goal setting and action planning is explained), module 3 and module 4.

### Video-coaching sessions

The video-coaching sessions will take place on alternate weeks to the modules (e.g., week 1 = module 1, week 2 = video-coaching, week 3 = module 2, etc.) through an online video calling program of participants’ choice. The coaching sessions will only be available for participants in the tailoring + video-coaching group. These participants will have a ‘video-coaching’ tab on the website which will include a link to free online video calling programs including Skype, Google Hangout, Yahoo Messenger and Face Time, and information on how to set up an account. The website will also provide a link to a calendar where participants can book their time slot with the Activity Coach. They will need to book a time for each of the 4 sessions, and will be asked to do this immediately following the completion of a module (thus one week in advance of the coaching session). During the session the Activity Coach will comment on the tailored advice participants received in the module from the previous week. The Coach will ask participants if they understood the advice, if they agree with the contents of the advice (and if not, why), if they have been able to act on the advice, and if they encountered any problems adhering to the advice. The coach will also ask participants if they have any questions. The coach will ensure that the video call will be a maximum of 15 minutes in length. The sessions are purposefully designed to be short to assess whether this method can be viable for future large scale interventions, and to keep the time requirements of participants to a minimum.

### Measures

Participants will receive a total of 4 questionnaires to assess their physical activity, the correlates of physical activity related to the Theory of Planned Behaviour and quality of life across 4 time points (baseline, immediately after the end of the intervention (week 9), at 6 months and at 12 months post-baseline). Participant’s demographics and satisfaction with the intervention will only be assessed in the baseline and post intervention (week 9) questionnaires respectively. The satisfaction questions will only be given to the intervention groups, as the wait-list control participants will not have completed the intervention at this time point (week 9). The individual measures included in the questionnaires are explained below. Participant engagement, participant retention, and video-coaching feasibility will be measured throughout the intervention. Video-coaching feasibility will be measured by participant satisfaction and fidelity of the video-coaching sessions, and intervention engagement will be measured through website user statistics and intervention fidelity which are explained in detail below.

#### Demographics

Participant’s demographics including gender, age, BMI, marital status, income, education, employment and location will be assessed in the baseline survey.

#### Physical activity

The validated Active Australia Questionnaire will be used to measure total physical activity and whether participants meet the physical activity guidelines [53]. This tool assesses the number of sessions and total time spent walking, participating in moderate physical activities, vigorous physical activities and gardening during the previous week. Total physical activity time is calculated by summing the time spent walking, performing moderate-intensity physical activity, and performing vigorous-intensity physical activity multiplied by two. Physical activity sessions need be 10 minutes or longer to be included. Participants are categorized as being sufficiently physically active for health benefits if they participated in a minimum of 150 minutes of physical activity per week. The Active Australia Questionnaire has been found to have a good test-retest reliability (Kappa = .52) [54], a high percentage agreement with other physical activity measures (67%-75%) [55] and is sensitive enough to detect changes in physical activity [14].

#### Quality of life

The SF-12v2 will be used to measure participant’s quality of life by assessing participants physical and mental health status. The SF-12v2 measures 8 health domains: physical functioning, role participation with physical health problems (role-physical), bodily pain, general health, vitality, social functioning, role participation with emotional health problems (role-emotional), and mental health [56]. A physical health component and a mental health component summary scores are calculated using norm based standardised scores. The SF-12v2 was developed as a short version of the SF-36, has been proven to be a valid and reliable measure of quality of life. It has good construct validity compared to other measures of quality of life including the SF-36 [PHC *r* = .95, MCH *r* = .96 [57]], and good test-retest reliability [PHC *r* = .89, MCH *r* = .76 [56]].

#### Correlates of physical activity related to the Theory of Planned Behaviour

Constructs of the Theory of Planned Behaviour including attitude, subjective norm, perceived behavioural control and intention towards physical activity will be measured using a 16 item questionnaire developed by R Rhodes, E, D Hunt Matheson and R Mark [58,59]. The measures for all constructs have shown good reliability (*α* = .80-.95) and attitude, perceived behavioural control and subjective norm have a good predictive validity of intention (*r* = .85) [58]. To measure attitude participants will be asked to respond to “For me, regular physical activity over the next 2 weeks would be. . . .” by selecting a response on six 7-point bipolar adjective scales that measure both instrumental (beneficial/harmful, useful/useless, wise/foolish) and affective (enjoyable/unenjoyable, interesting/boring, relaxing/stressful) aspects of attitude. Subjective norm will be measured by 4 items on a 7-point Likert scale, for example “Most people who are important to me would encourage me to engage in regular physical activity over the next 2 weeks”. Perceived behavioural control will be measured by three items on a 7-point Likert scale, for example “In the next 2 weeks, doing physical activity, if I really wanted to, is under my control”. Intentions will be measure by 3 items on a 7-point Likert scale, for example “I am committed to engage in physical activity over the next 2 weeks”, A 4 item planning scale will also be used to assess the plans participants have to increase their physical activity. The planning scale was developed by L Trinh, RC Plotnikoff, RE Rhodes, S North and KS Courneya [60], and includes 4 items, ‘I have made plans concerning ‘when’, ‘where’, ‘what’ and ‘how’ I am going to engage in regular physical activity in the coming month’. The items will be assessed on a 7-point Likert scale with options ranging from ‘no plans’, to ‘detailed plans’. L Trinh, RC Plotnikoff, RE Rhodes, S North and KS Courneya [60] developed this scale based on the guidelines by I Ajzen [59], and found it to explain a significant percentage of the variance in physical activity behaviour (*r* = .50; *p* < .001).

#### Participant satisfaction

Intervention satisfaction will be assessed for intervention group participants only. Participants’ satisfaction with different parts of the intervention will be assessed by a questionnaire (68 items) that was specifically developed for this study, though based on previous research [61] and will include items on the questions needed to generate the personalised feedback, the tailored advice, website usability, the coaching sessions (for tailoring + video-coaching participants only) and the overall satisfaction with the program. The majority of items are on a 5-point Likert scale where participants are asked to rate their agreement (strongly agree to strongly disagree) to statements about the intervention, for example, ‘the questions were easy to understand’. Four open ended items will also be included in the sections on the tailored advice, website usability, the coaching session and the overall program to provide participants with the opportunity to describe 1) what they liked, 2) what they didn’t like, 3) any recommendations they have to improve the program and 4) if they have any further comments.

#### Website user statistics

Website user statistics will be collected for each participant. These will be measured by google analytics software, and include number of website visits, average number of pages viewed during a visit, and average visit duration during the 8 week intervention period and during the 12 month post intervention period leading up to the follow up questionnaires.

#### Intervention fidelity

To determine whether the intervention was delivered as planned, participant’s completion of the intervention surveys, and time of completion (whether or not they were completed on time) will be recorded. The coaching participant’s completion of the coaching sessions, the length of the coaching sessions, and topics covered in the coaching sessions will also be recorded to measure intervention fidelity.

### Statistical analyses

#### Intervention effects

Data will be analysed using intention-to-treat principles. Physical activity will be modelled using the using linear mixed models with random intercepts, the fixed effects of group (control, tailoring only, tailoring + video-coaching) and time (baseline, post-intervention, 6-months, 12-months), and a group by time interaction and will adjust for potential confounders including gender, age, education, income, employment, location, marital status and BMI if they are associated with physical activity and time.

#### Secondary analyses

The secondary analyses will be conducted using linear effects modelling to determine the effect of group and time on Theory of Planned Behaviour constructs and quality of life. Linear mixed modelling will also be used to compare retention, satisfaction, intervention fidelity and website user statistics between groups. Multiple regression analyses will be conducted to assess Theory of Planned Behaviour concepts including intention, attitude, subjective norm, perceived behavioural control and planning as mediators for physical activity changes. Multiple regression analyses will also be used to asses these Theory of Planned Behaviour concepts as well as demographic variables (age, gender, income, marital status, education and BMI) as moderators for physical activity changes. Descriptive statistics will be used to assess participant satisfaction and fidelity of the video-coaching session.

### Sample size

The sample size needed to detect between group differences in physical activity levels across the primary time points (baseline and post-intervention) through linear mixed models was calculated from the sample size analysis developed by K Lu, X Luo and P Chen, Y [62]. The alpha level was set to ≤0.05 (80% power). The effect size was estimated to be small (.43) based on the findings from a recent meta-analysis looking at the effectiveness of physical activity interventions with a minimal control group [12]. Reviews and meta-analyses have found average attrition levels of web-based physical activity levels to be around 25% [12,13]. Therefore an estimated attrition of 25% was factored into the calculations. The analysis revealed that a sample size of 300, or 100 in each study arm, is required for the current study to detect small effects between group differences in physical activity across the two time points.

## Discussion

More research is needed to determine effective combinations of web-based intervention components to improve intervention effectiveness in terms of participant engagement and long-term behaviour changes [12]. An understanding of effective low cost methods of delivering personalised physical activity advice (online coaching and tailored advice) is important as, although there is some evidence for the effectiveness of both components [18,20,23], each form of personalised advice has different benefits and costs. Web-based interventions commonly use computer-tailored advice as it can deliver similar content at a lower cost than coaching sessions [18,20]. However coaching adds a social support element that is found to improve intervention outcomes [22,23]. The current study will measure the effectiveness of a novel approach, combining both computer-tailored advice and an online coaching session using a video-calling program (eg, Skype) in order to provide participants with an element of social support, and at a low-cost through minimising the content the coach is required to deliver and utilising the availability of free online video-calling programs. The physical activity, engagement, retention and satisfaction outcomes of brief online coaching sessions in addition to a web-based physical activity intervention that provides computer-tailored advice will be assessed. The findings will shed light on whether this new approach to delivering tailored advice is feasible, and more effective than stand-alone computer-tailored advice. Knowledge of the effectiveness of brief online coaching sessions will be beneficial for the development of future web-based physical activity interventions that can be delivered at a large scale and are effective at engaging participants and producing long-term behaviour changes.

## Abbreviations

AAQ: Active Australia questionnaire; TPB: Theory of planned behaviour; ELM: Elaboration likelihood model.

## Competing interests

The authors declare that they have no competing interests.

## Authors’ contributions

SA conceived the study, drafted the manuscript and will carry out the proposed protocol. CJ, RP and CV played a significant role in establishing the study design and drafting the manuscript. All authors read and approved the final manuscript.

## Pre-publication history

The pre-publication history for this paper can be accessed here:

http://www.biomedcentral.com/1471-2458/14/738/prepub
